# Retrospective study of immediate postoperative electron radiotherapy for therapy-resistant earlobe keloids

**DOI:** 10.1007/s00403-019-01922-z

**Published:** 2019-04-30

**Authors:** Chun-Lei Liu, Zhi-Yong Yuan

**Affiliations:** 1 Tianjin Medical University Cancer Institute and Hospital, National Clinical Research Center for Cancer, Key Laboratory of Cancer Prevention and Therapy, Tianjin, Tianjin’s Clinical Research Center for Cancer, Tianjin, 300060 China; 2 Chifeng Municipal Hospital, Chifeng Clinical Medical School of Inner Mongolia Medical University, Chifeng, 024000 China

**Keywords:** Keloid, Electron radiotherapy, Three-fraction, Earlobe

## Abstract

Keloid resection followed by adjuvant radiotherapy is the most efficacious treatment for keloids. However, for earlobe keloids, an optimal protocol for the total dose and fractions of adjuvant radiation has not yet been established. We retrospectively analyzed the efficacy and safety of immediate three-fraction electron radiotherapy after operation for resistant earlobe keloids. From 2011 to 2017, three-fraction electron radiotherapy with single dose of 5 Gy was given postoperatively to 23 patients with 30 keloids in our hospital. The first fraction of adjuvant radiotherapy was administered within 2 h of surgery, and the other two sessions were completed within the next day or two. Five (16.7%) primary keloids and 25 (83.3%) recurrent keloids were examined in this study. The primary endpoint was the local control rate, which was 86.7% after a median follow-up of 26 months (14–93 months). Secondary endpoints were acute and late procedure-related complications, and no severe complications were observed after combination therapy. Our results suggest that three-fraction electron radiotherapy after excision within 2 days of surgery is a safe and effective protocol for the prevention of earlobe keloid recurrence that can also improve patient compliance and comfort.

## Introduction

Keloids are a benign fibro-proliferative disease that spread beyond the original borders of a wound in response to an injury or trauma. Earlobe has been mentioned as the most common location for keloids after any injury, most commonly cosmetic piercing [1], and due to the unique characteristics of the earlobe, such as its free edge, arc-like shape, and lack of cartilage. The incidence of earlobe keloids after ear piercing has been estimated at 2.5% [25]. It often causes pain, pruritus, and tenderness, and can cause cosmetic disfigurement, impairing the quality of life. Therefore, various treatment regimens including surgery, corticosteroids injection, cryosurgery, laser therapy, and radiotherapy are recommended for keloids [6, 11, 19, 24]. Any monotherapy is avoided because of the limited success of a single treatment. For example, the recurrence rate after surgical excision alone is as high as 80% [2, 4, 7]. At the morphological level, human earlobes have an intricate anatomical structure and the average length is approximately 2 cm [3]. Extralesional surgical excision in combination with other treatments, especially adjuvant postoperative radiotherapy, is considered to be the most efficacious protocol for resistant keloids, reducing the relapse rate to below 10% and improving patient convenience [13, 20]. However, there is no consensus on the optimal radiation dose and fractionation schedule, especially for external radiotherapy. In this study, we retrospectively evaluated the results of surgical excision combined with immediate adjuvant radiotherapy in patients with therapy-resistant keloids.

## Methods

### Patient characteristics

From January 2011 to July 2017, clinical data on 23 patients (8 males and 15 females) with 30 keloids of the earlobe were retrospectively reviewed, and these 23 patients were treated with surgical excision followed by immediate adjuvant electron radiotherapy. The median age of the patients undergoing treatment was 28 years (17–63 years). Four patients had never undergone any prior treatment to manage their earlobe keloids, because they also had keloids on their faces and other parts of their bodies. Patients in this situation are considered to be more likely to relapse after treatment. Other keloids had already received one or more pretreatments; that included surgical resection, corticosteroid injection, cryotherapy, Y-laser, and a combination of these treatments. All patients consented to the treatment protocol.

### Surgical treatment

To receive adjuvant radiotherapy in time after surgical resection, all patients made an appointment in advance. To accurately determine the range of target for radiotherapy, surgeons need to work together with radiation oncologists to mark the locations of earlobe keloids.

Extralesional excision was performed in all keloids with local anesthesia using xylocaine 1% with adrenalin 1:100,000. Hemostasis was achieved with electrocautery, and the wounds were closed without tension. No hyper-proliferative core excision and keloids flaps were performed. All excised specimens underwent independent histologic analysis to determine the diagnosis. The patients were transferred to the Department of Radiation Oncology to develop a radiotherapy plan and receive the first treatment within 2 h of excision. Seven days after radiotherapy, the skin sutures were removed.

### Radiotherapy

**Fig. 1:**
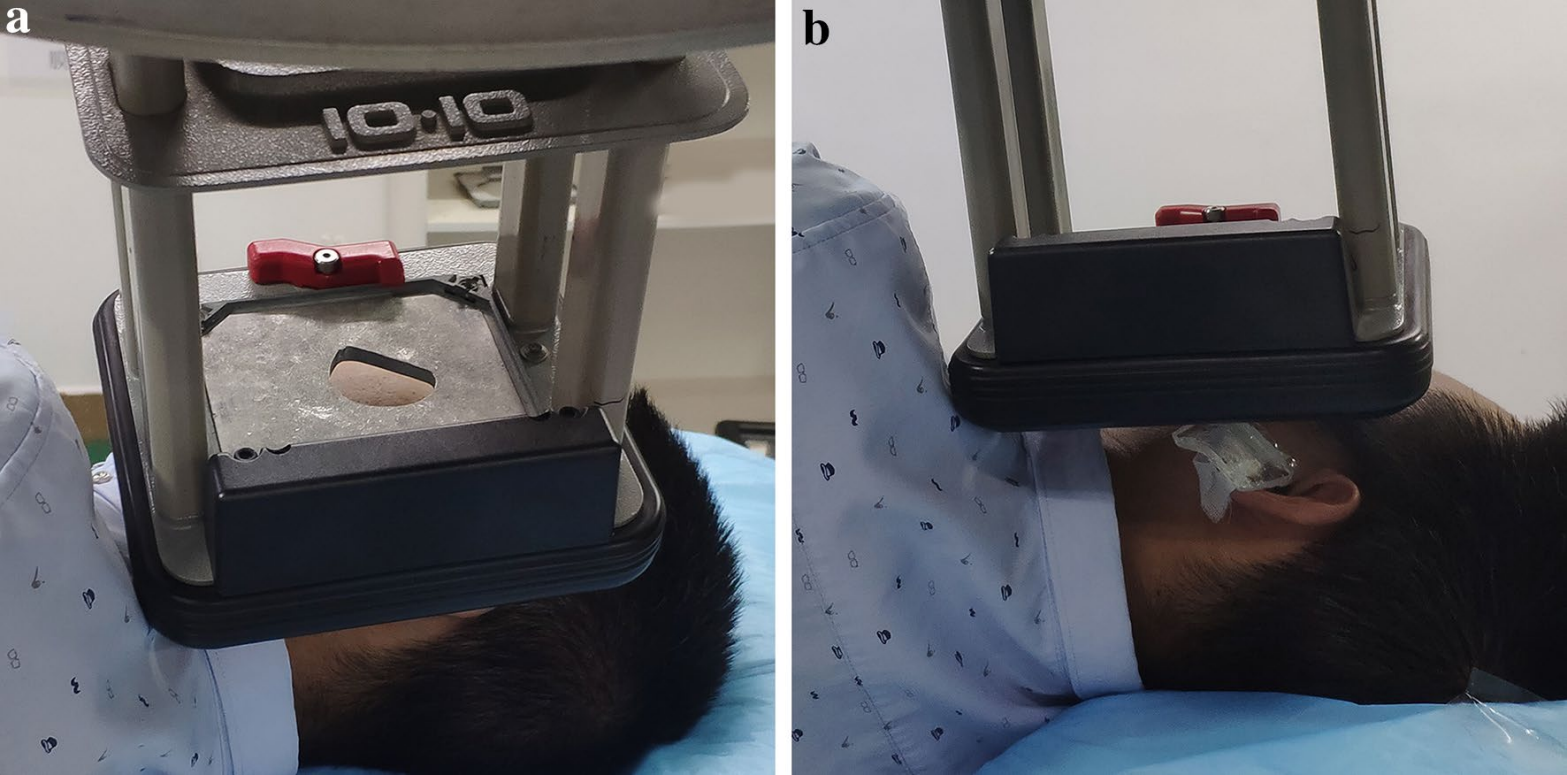
Shield effect on dosimetry: **a** shows the lead shielding to protect the surrounding normal tissue; **b** shows a 0.5 cm wax to increase the surface dose

Adjuvant postoperative radiotherapy for all keloids was implemented within 2 h of surgical excision using a 6-MeV electron-beam, with full shielding to protect the normal tissues (Fig. 1). Before radiotherapy, radiotherapy oncologists applied 0.5 cm wax to increase the surface dose. Previous keloid cases were treated with a total radiation dose of 15 Gy that was delivered in three fractions over 3 days that was initiated on the same day as surgery. Since 2015, we have shortened the course of radiotherapy from 3 days to 2. The remaining two fractions of radiotherapy were given on the second day after surgery, and must be at least 6 h apart.

### Follow-up

Patients were required to return to the hospital regularly for a face-to-face follow-up. If some patients could not return for an in-office follow-up, they were contacted by phone to confirm the success of the keloid treatment and were asked to provide pictures. Follow-up visits were scheduled at 3 months, 6 months, 1 year, and every year thereafter, as follow-up visits should extend for at least 1 year after treatment to improve the validity of the outcome, because only after that period is the chance of recurrence minimal [8, 29]. The primary endpoint was the local control rate. The secondary endpoints were acute and late procedure-related complications according to the Common Terminology Criteria for Adverse Events version 4.0 (CTCAE v4.0). Acute toxicity occurred within 3 months after radiotherapy, and late toxicity occurred within 3–6 months or longer. Recurrence was defined as the presence of a new keloid scar in the treated location, with sustained growth, and classic symptoms such as pain, and itching. A hypertrophic scar was defined by raised scars that did not grow beyond the boundaries of the original wound.

## Results

**Fig. 2:**
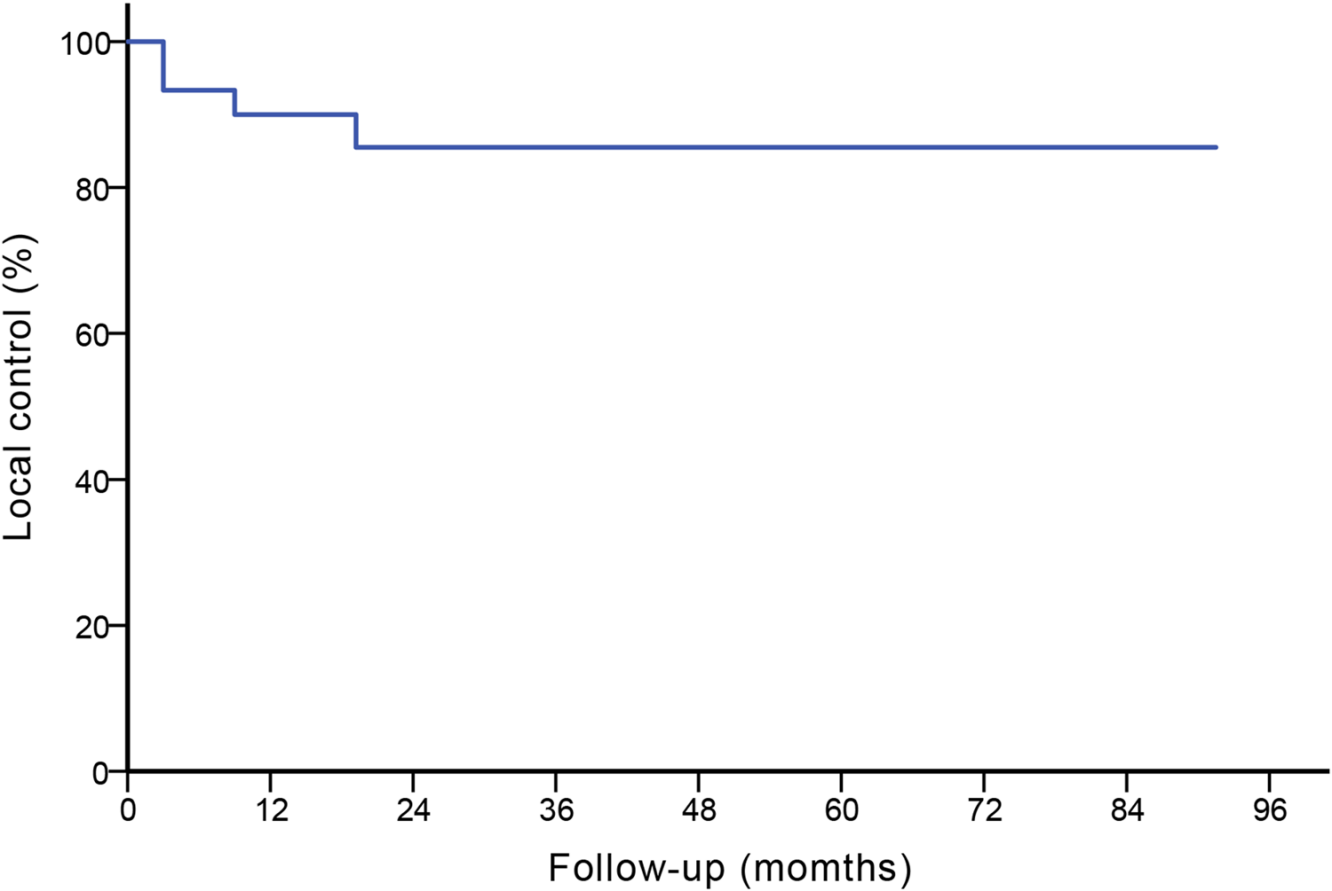
The local control and follow-up states of all 30 keloids: the 5-year Kaplan–Maier estimated local control was 86.7%

Twenty-three patients with 30 keloids were entered into the study, including 5 (16.7%) primary keloids and 25(83.3%) recurrent keloids. No keloids had been previously treated with any form of radiotherapy. All the patients were followed up and the result is shown in Fig. 2. Other patient and keloids characteristics are shown in Table 1.

**Table 1:** Summary of patient and keloid characteristics

Characteristic	No. (%)
Gender	
Male	8 (35%)
Female	15 (65%)
Median age (range)	28 (17–63)
Anatomical location	
Unilateral	16 (69.6%)
Bilateral	7 (30.4%)
No. of keloids	30
Left ear/right ear	16 (53.3%)/14 (46.7%)
Cause of keloids	
Ear piercing	21 (70%)
Acne	6 (20%)
Trauma	3 (10%)
Previous treatment	
None	4 (13.3%)
Surgery alone	10 (33.3%)
Cryotherapy	2 (6.7%)
Laser treatment	3 (10.0%)
Combination	11 (36.7%)
Maximum diameter of lesion (cm)	2.3 (1.4–3.7)
Duration of radiotherapy	
Three days	6 (20%)
Two days	24 (80%)
Radiotherapy schem	15/(3 × 5 Gy)
BED (Gy) (*α*/*β* = 10)	22.5

At the last follow-up date, 4 (13.3%) keloids had recurred after a median follow-up of 26 months (14–93 months). Two of the lesions were in the same patient and recurred 3 months after radiotherapy. Two other keloids relapsed 9 and 19 months after radiotherapy, respectively.

**Fig. 3:**
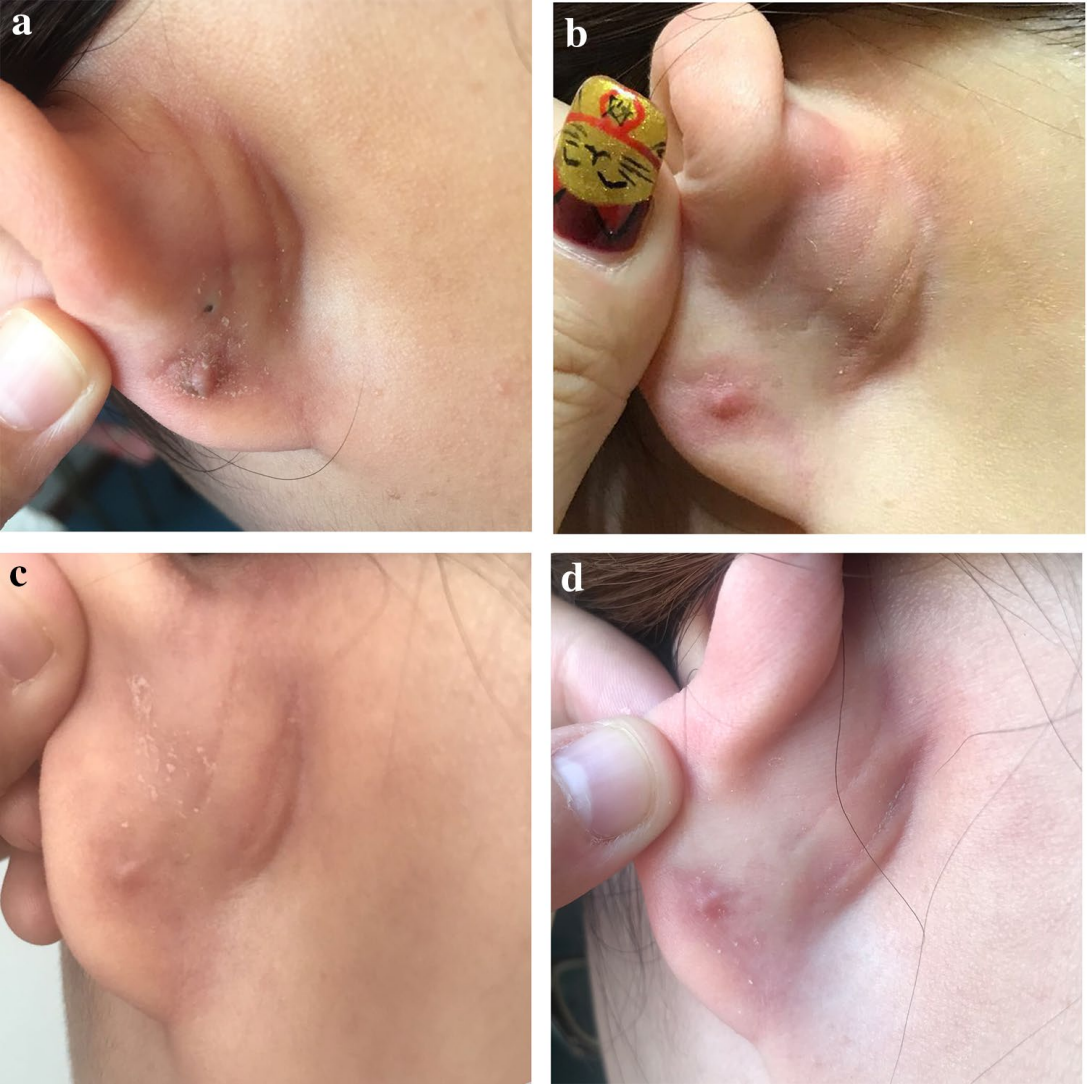
A typical earlobe hypertrophic scar after treatment. **a**–**d** Three months, 6 months, 12 months, and 24 months after postoperative radiotherapy, respectively

One patient experienced peeling and slight hypertrophied scars (Fig. 3). The pretherapeutic symptoms were completely resolved on other patients without recurrence. There were no acute adverse effects such as delayed wound healing, skin ulceration, or infections. Four keloids in three patients showed mild hyperpigmentation, and no other serious late side effects, such as skin atrophy, telangiectasia, and subcutaneous fibrosis were noted by the physicians who performed the examination.

## Discussion

Keloids are benign, fibro-proliferative lesions that may result in cosmetic deformities as well as decrease quality of life [8, 28]. Patients seek treatment to eradicate symptoms such as itching, pain, and burning; that cause the largest burden in keloid disease [20, 22, 24]. Adjuvant irradiation using electron radiotherapy or brachytherapy for keloids has been used for nearly a century and achieved an excellent therapeutic effect, especially for earlobe keloids (Table 2). However, to date, no widespread consensus of the optimal radiation regimen to prevent recurrence and obtain the best treatment results has been established.

**Table 2:** Summary of results of adjuvant postoperative radiotherapy for earlobe keloids

References	No. of patents	No. of keloids		Total dose (Gy)	Fraction scheme (Gy)	BED(Gy) (*α*/*β* = 10)	Median follow-up (range) (Mos)	LC rate (%)	S and R interval
HDR brachytherapy									
Garg et al. [10]	12	Earlobe	17 8	15	3 × 5	22.5	26 (12–71)	88.2 87.5	24 h
Arneja et al. [1]	25			15	3 × 5	22.5	35 (mean) (24–57)	92	1 h
Van et al. [27]	28	Earlobe	32 37%	12	2 × 6	19.2	33.6	96.9 –	4 h
Hafkamp et al. [12]	24	Ear	29 14	13	1 × 13	29.9	53 (19–95)	75.9 85.7	2 h
Jiang et al. [14]	29		37	18	3 × 6	28.8	49.7 (7.9–91.9)	91.9	6 h
Electronic radiotherapy									
Present study	23	Earlobe	30	15	3 × 5	22.5	26 (14–93)	86.7	2 h
Ogawa et al. [20]	39 106		47 127	15 10	3 × 5 2 × 5	22.5 15	18	95.7 94.5	–
Song et al. [26]	12	Earlobe	16 8	10	1 × 10	20	20 (mean)	100 100	72 h
Vila et al. [30]	19	Earlobe	20 10	15	3 × 5	22.5	40 (12–68)	76.5 80	4 h
Kim et al. [16]	27	Earlobe	13 25	12 15	3 × 4 3 × 5	16.8 22.5	132 (132–106) 49.3 (15–124)	76.9 93.7	24 h
Shen et al. [24]	568	Earlobe	834 239	18	2 × 9	34.2	40 (12–160)	88.3 96.7	24–48 h

*BED* biologic equivalent dose, *Mos* months, *LC* local control, *S* surgery, *R* radiotherapy, *HDR* high dose rate

In recent years, there have been extensive studies using interstitial brachytherapy for the postoperative treatment of keloids. Ping Jiang et al. [14] reported that, after a median follow-up of 49.7 months, they obtained a 92% local control rate using perioperative interstitial high-dose-rate (HDR) brachytherapy in 29 patients with 37 recurrent keloids. Eveline Bijland et al. [5]. treated 238 keloids using three different HDR brachytherapy schemes including 2 × 9 Gy, 3 × 6 Gy, and 2 × 6 Gy, and observed an overall local control rate of 91.7% after at least 12 months of follow-up. Although few recent studies have focused specifically on external radiotherapy, good result has been reported. Song et al. treated 12 patients using electron-beam radiotherapy with a single fraction of 10 Gy [26]. No recurrence was found after a mean follow-up period of 20 months. A recent analysis reported a 96% local control rate for 145 patients with 174 lesions [20]. The schemes of postoperative radiotherapy, using a 4-MeV electron-beam, included 15 Gy administered in three fractions and 10 Gy administered in two fractions. In the present research, patients with earlobe keloids at our hospital were treated with surgical excision followed by 6 MeV electron-beam radiotherapy. After a median 26-month follow-up, we observed local control in 86.7% cases, which is consistent with that reported in other studies.

Although combining surgical excision with adjuvant radiotherapy is effective, the optimal biologic equivalent dose (BED) for the inhibition of keloid recurrence is uncertain. In the literature, the *α*/*β* value of the skin was mostly reported to be 10. Kal et al. [15] conducted a dose–response analysis of external-beam radiotherapy for keloids and recommended that the BED should be at least 30 Gy to achieve a recurrence rate of < 10%. Another meta-analysis and systematic review including 72 studies reviewed radiation-based treatments used for keloid management [18]. To achieve 90% control of keloid recurrence, they suggested a dose of more than 35 Gy for external-beam radiotherapy according to the dose–response curve. In contrast, as for brachytherapy, the BED was recommended to be approximately 20 Gy, which is sufficient. To obtain a similar risk of recurrence, Eveline Bijland et al. [5] compared three different treatment protocols. The BED could be as low as 19 Gy, but they also predicted that this dose may be the lower limit of effectiveness for the prevention of keloids. Our present results indicated that similar local control rates could be reached with a BED less than 30 Gy using electron-beam radiotherapy. In regard to different radiation modalities, no statistically significant difference was found in terms of local control between brachytherapy and electron-beam irradiation [18]. However, calculations of the BED for different radiation modalities are diverse. For brachytherapy, the algorithm of equivalent dose in 2 Gy fractions (EQD2) is an extensive application in the clinic. Further randomized controlled studies should be carried out to determine the essential biologic equivalent dose for keloid management.

A short interval time from surgery to the initiation of adjuvant radiotherapy is currently recommended. Van Leeuwen et al. [28] conducted a systemic review and found that a short-time interval of less than 7 h between keloid excision and irradiation resulted in a lower recurrence rate than that observed with longer time intervals of more than 24 h. Ping Jiang et al. [14] administered the first fraction using brachytherapy within 6 h and observed a very low risk of recurrence. We compressed this interval to less than 2 h, which is in line with previous report [12]. However, Enhamre and Hammar [9] found that the treatment success had nothing to do with the time interval between surgical resection and radiotherapy. Sakamoto et al. [23] stated that the time interval between surgical resection and adjuvant radiotherapy did not affect local control and adverse effects. The true mechanism for this remains unclear and controversial. Many studies have suggested that radiation can prevent keloidal fibroblasts from proliferating [15, 17]. This seems illogical, as surgical excision removes all keloid fibroblasts. Another explanation could be that after keloid resection, some types of cells such as collagenocyte and fibroblast that play an important role in keloid formation in rapidly proliferate. From a radiobiological point of view, these cells should be radiosensitive, at least theoretically. As this process begins directly after the operation, it is important to start irradiation as quickly as possible by transferring the patient immediately after surgery to the radiation department. Therefore, when radiotherapy should be initiated after excision to obtain a better prognosis is yet to be investigated.

The complication results in our study indicated that radiotherapy following surgical excision is a safe treatment modality. Electron-beam radiotherapy has routinely been used for the treatment of keloids in our hospital to reduce radiation damage to the surrounding normal tissues. Although the majority of patients were concerned about carcinogenesis from irradiation, no reports suggested that postoperative radiotherapy can induce a malignant neoplasm in the earlobe after irradiating for keloids [21]. The radiotherapy dosage used in the present study was 15 Gy administered in three fractions, and the BED is 22.5 Gy, which is lower than the proposed BED of more than 30 Gy [29]. However, further prospective studies should be carried out, and longer follow-up is necessary.

It should also be noted that our study was not without limitations. First, similar to most previous studies, the number of patients included in the present study was too small (only 30 keloids). Second, most patients have previously received a variety of treatments, and the impact of these pretreatment on the outcomes is difficult to assess. Third, fewer than half of the patients returned to the hospital regularly for scheduled follow-ups, which may have led to bias in the evaluation of efficacy. Nevertheless, the major finding of our study was that not only good local control but also low acute and long-term toxicity were observed with this treatment regimen. In particular, infection complications were avoided.

## Conclusion

In summary, immediate postoperative radiotherapy using a total of 15 Gy in three fractions was well tolerated and received without any adverse effects. This was a safe and effective protocol for the prevention of earlobe keloid recurrence. Because the treatment time was reduced from 3 days to 2 days, patients completed treatment in the outpatient department, which avoided inpatient inconvenience and increased patient compliance. Further prospective research is needed to prove the benefits and advantage of this keloid treatment regimen.
